# Cell Cycle Regulation by Heat Shock Transcription Factors

**DOI:** 10.3390/cells11020203

**Published:** 2022-01-08

**Authors:** Yasuko Tokunaga, Ken-Ichiro Otsuyama, Naoki Hayashida

**Affiliations:** 1 Division of Molecular Gerontology and Anti-Ageing Medicine, Department of Biochemistry and Molecular Biology, Graduate School of Medicine, Yamaguchi University, Ube 7558505, Japan; yako1229@yamaguchi-u.ac.jp; 2 Department of Laboratory Science, Graduate School of Medicine, Yamaguchi University, Ube 7558505, Japan; otsuyama@yamaguchi-u.ac.jp

**Keywords:** cell cycle, HSF1, HSF2, cell cycle arrest, APC/C complex

## Abstract

Cell division and cell cycle mechanism has been studied for 70 years. This research has revealed that the cell cycle is regulated by many factors, including cyclins and cyclin-dependent kinases (CDKs). Heat shock transcription factors (HSFs) have been noted as critical proteins for cell survival against various stresses; however, recent studies suggest that HSFs also have important roles in cell cycle regulation-independent cell-protective functions. During cell cycle progression, HSF1, and HSF2 bind to condensed chromatin to provide immediate precise gene expression after cell division. This review focuses on the function of these HSFs in cell cycle progression, cell cycle arrest, gene bookmarking, mitosis and meiosis.

## 1. Introduction

The mitosis phenomenon was discovered over one hundred years ago, and many scientists have performed experiments to make novel discoveries. In the early 20th century, germ cells were the predominant focus of studies and the change in cell morphology during mitosis was widely known among researchers [1,2]. Important studies were performed all over the world and many essential findings were reported [3,4,5,6,7,8]. Changes in cytoplasmic structures were also revealed in studies that took place over a short period, and structural changes of mitochondria, Golgi substance, new cell walls, mitotic spindles, and centromere were identified [9,10,11,12,13,14]. Classification into Gap1 (G_1_), synthesis (S), Gap2 (G_2_), and mitosis (M) phases was suggested following the observation of cytoplasmic structures [15,16,17,18,19,20,21,22,23,24].

In the early 1970s, Leland Hartwell and his colleagues discovered several genes responsible for the eukaryotic cell cycle in budding yeast (*Saccharomyces cerevisiae*) (reviewed in [25]). In 1970, they discovered three genes: *cdc-1*, *cdc-2*, and *cdc-3* (*cdc* stands for “cell division cycle”) [26]. Several years later, it was revealed that *cdc-1* encodes metallophosphodiesterase, *cdc-2* encodes the DNA polymerase delta catalytic subunit and *cdc-3* encodes septin family members [25]. Hartwell and his colleagues examined approximately 1500 temperature-sensitive mutants and isolated 148 mutants. They characterized these yeast mutants and succeeded in determining the locations of 14 genes on the yeast genetic map [27]. Subsequently, they found that four genes, including *cdc-4*, *cdc-7*, and *cdc-28*, are required for the initiation of yeast DNA synthesis and discovered that the *cdc-28* gene is required for DNA synthesis and budding [28]. In the *cdc-28* mutant, cells arrested as unbudded cells, and the cell cycle was also arrested before the initiation of DNA synthesis. Due to the importance of *cdc-28* in cell division, *cdc-28* became the first cloned cell cycle gene [29].

Discoveries by Hartwell and his colleagues contributed to cell division and cell cycle research and the progression of other researchers’ studies. Their research became a pioneering study.

Paul Nurse and his colleagues also made valuable discoveries. They used temperature-sensitive mutants of the fission yeast *Schizosaccharomyces pombe*, different from the budding yeast *Saccharomyces cerevisiae* that Hartwell used, and found that their mutants have genetic mutations involved in cell size control over DNA synthesis and a second control acting on nuclear division [30]. Moreover, they discovered that *cdc-2* gene product activity is important for determining when mitosis takes place and is required for starting and controlling mitosis. They also successfully made a genetic map of the *cdc-2* locus and its isolation [31,32]. The understanding of *cdc-2* in the cell cycle was advanced by Nurse and his colleagues.

While Leland Hartwell and Paul Nurse made many discoveries using yeast, Tim Hunt examined protein synthesis using fertilized sea urchin eggs. Hunt and his colleagues analyzed the pattern of protein synthesis before and after fertilization using a two-dimensional gel separation technique and found that the pattern of expressed proteins was changed [33]. They first noticed that one of these proteins is destroyed every time the cells divide. Then, they performed protein analysis experiments using ^35^S-methionine-added egg suspensions and discovered that some proteins start to be synthesized after fertilization and are destroyed at certain points in the cell division cycle. They examined this protein in detail and proposed to call these proteins “cyclins” [33]. Importantly, they also showed data indicating the existence of multiple cyclins and described two cyclins, cyclin A and cyclin B, discovered from the clam *Spisula solidissima* in this paper.

After the discovery of cyclins, molecular cloning was succeeded very soon. Ruderman and his colleagues cloned cyclin A from Xenopus oocytes [34]. Hunt’s group also reported the cloning of the cyclin from sea urchin eggs [35].

Leland Hartwell, Paul Nurse, and Tim Hunt were awarded a Nobel Prize in 2001, and the importance of cell cycle regulation has been more widely recognized among researchers in various fields. It is notable that the essential genes in the cell cycle were discovered through experiments using temperature-sensitive mutant yeast cells. Furthermore, the most famous transcription factor against temperature stress is heat shock transcription factor/heat shock factor (HSF).

HSF was discovered as a transcription factor essential for the heat shock response. The heat shock response is a cellular protective mechanism against heat stress that was discovered by Ritossa in 1962. While he was keeping larvae of *Drosophila melanogaster* at 25 degrees, he observed that the salivary gland of *Drosophila* was puffing when under additional heat stress at 37 degrees, which is a sign of enlarged chromosomal formation and indicates that some specific mRNA synthesis was accelerated [36,37,38]. Subsequently, Tissières and his colleagues showed that accelerated mRNA synthesis occurs in the heat shock protein (HSP) genes [39,40].

**Figure 1:**
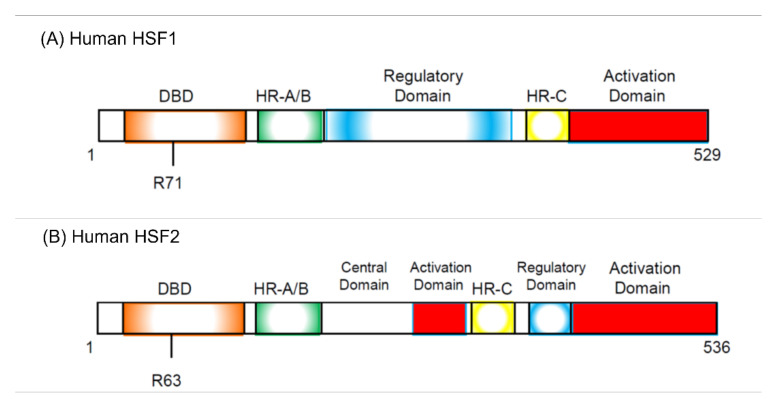
Structure of human HSF1 (hHSF1, (**A**)) and human HSF2 (hHSF2, (**B**)). hHSF1 and hHSF2 have similar domain structures, but several differences exist. R71 and R63 are required for DNA binding in hHSF1 and hHSF2, respectively. The regulatory domain suppresses trimerization of both HSFs and inhibits transcriptional activation. Trimerization is required for the activation of HSFs. DBD, DNA-binding domain; HR-A/B, hydrophobic heptad repeats A and B; HR-C, C-terminal hydrophobic heptad repeat.

Five years before the discovery of HSF, it was reported that there are some proteins that specifically bind to the heat shock element (HSE) sequence commonly found in the HSP promoter following in vitro and in vivo experiments performed by Wu’s group [41,42]. Several research groups discovered HSF protein from *Saccharomyces cerevisiae*, *Drosophila melanogaster*, and HeLa cells in 1987 [43,44,45,46]. In 1991, two HSFs, HSF1 and HSF2, were cloned in humans and mice [47,48,49]. HSF1 is activated by heat stress, but HSF2 was found to be activated first by hemin [50]. As two HSFs were cloned at the same time, the previously discovered HSF was called HSF1, and the other HSF was called HSF2 (Figure 1).

As HSF1 was discovered to be a transcription factor protecting cell survival through the heat shock response, the HSF1–HSP pathway was exclusively studied in the early period. However, as already described, the essential genes in the cell cycle were discovered in experiments using temperature-sensitive mutant yeast cells. Interestingly, other experiments using temperature-sensitive mutant yeast cells opened the door to the study of the cell cycle and HSFs.

**Significance in** **Section 1:**

- The essential genes in the cell cycle were discovered through experiments using temperature-sensitive mutant yeast cells.
- HSFs were discovered as proteins specifically binding to HSE sequence commonly found in the *HSP* promoter.

## 2. Discovery of HSF Involvement in Cell Cycle Regulation

The first evidence that HSF1 is involved in cell cycle regulation came from experiments using mutant yeast cells. Smith and Yaffe found that yeast cells containing a mutation in the *mas3* gene display retarded progression through the G_2_ stage, and that this *mas3* gene encodes HSF1 [51]. In addition, *mas3* cells showed that the induction of a major heat shock gene, *SSA1*, is defective under heat stress at 37 degrees. These are the first data indicating that HSF1 mediates cell cycle progression.

After this report, other laboratories published papers similarly indicating HSF1′s mediation of the cell cycle. Thiele and his colleagues discovered that yeast cells bearing a truncated form of HSF1 in which the C-terminal transcriptional activation domain is deleted undergo reversible cell cycle arrest under heat stress at 37 degrees in the G_2_/M phase and exhibit a marked reduction in HSP90 expression [52]. Using wild-type (WT) mouse embryonic fibroblasts (MEFs) and HSF1-deficient MEFs, Dix and his colleagues discovered that a three-fold increase in G_2_/M phase-arrested cells occurred in WT–MEFs but not in HSF1-deficient MEFs after conditioning heat stress (43 degrees, non-lethal temperature) followed by lethal heat stress (45 degrees) [53]. However, Li and Martinez reported that human colon cancer HCT116 cells lacking HSF1 did not show G_2_/M arrest, and checkpoint activation was lost in these cells [54]. They also showed that p53 is involved in this phenomenon and that the relationship between HSF1 and p53 is critical for cell cycle regulation.

Calderwood and his colleagues reported that overexpression of HSF1 increases the proportion of G_1_ cells in HeLa cells under non-heat-stressed conditions [55]. Surprisingly, they observed that HSF1 bound to HSE sequence during the early G_1_ phase in the absence of heat stress. In addition, He and Fox reported the behavior of HSF1 during the cell cycle under non-heat-stressed conditions [56]. They measured the binding of HSF1 to the HSE with a gel mobility shift assay using cell extracts from Hoechst 33342-labeled heated Chinese hamster ovary (CHO) cells from the G_1_, S, and G_2_/M phases. Their study indicated that the binding activity of HSF1 is two-fold higher in the S phase than in the G_1_ or G_2_/M phases, but the HSF1 expression levels do not vary throughout the cell cycle [56]. Gross’ group also examined HSF1 behavior throughout the cell cycle using their original dinucleosomal heat shock promoter model and found that activated HSF1 cannot bind to DNA in G_1_-arrested cells but can bind following release from G_1_ arrest or after the imposition of either an early S- or late G_2_-phase arrest [57]. Their discoveries showed that HSF1 binding activity to the DNA of the target genes changes throughout the cell cycle and is related to G_1_, S, and G_2_/M phase arrest.

The existence of a relationship between HSF1 and the cell cycle was widely recognized among HSF researchers. Thus, some considered the possibility that HSF1 plays a role in cancer. The first evidence demonstrating this possibility was shown by a study using prostate carcinoma cell lines and carcinomatous prostate tissue sections from patients. In a comparison between the non-metastatic human prostate carcinoma cell line PC-3 and the metastatic variant PC-3M, higher expression of HSF1 was found in both mRNA and protein [58]. Moreover, HSF1 protein was expressed more highly in tumor tissues than in normal sections from the same patient. Calderwood and his colleagues also used PC-3 cells and stably expressed dominant-negative HSF1 (DN-HSF1), which lost transcriptional activities in the cells. In DN-HSF1-expressed PC-3 cells, aneuploidy was inhibited and cyclin B1 degradation was retarded [59], the latter being a key step in the control of mitosis. In addition, p21 expression was increased in DN-HSF1 cells. Although PC-3 is a p53-null cell, DN-HSF1 can induce p21 expression directly or indirectly [59]. The studies using prostate carcinoma cells and sections suggested that HSF1 can positively and negatively regulate cell cycle progression.

The mechanism by which HSF1 is involved in cell cycle regulation has been examined by many researchers. HSF1 was discovered to be a protein binding to HSE sequence found in the promoter of HSPs, the major and heat-inducible targets of HSF1 [43,44,45,46], but whether this HSF1–HSP pathway has a pivotal role in cell cycle regulation or another HSF1 pathway is more important was uncertain. At that time, new findings were provided instead by HSF2 research.

**Significance in** **Section 2:**

- Mutant HSF1 affects cell cycle progression and arrest.
- Higher expression of HSF1 is related to cancer and aneuploidy.

## 3. Bookmarking Is the Important Role of HSFs

It was already shown that HSF2 can bind to the *HSP70* promoter, but the reason remained unclear [60,61,62]. Sarge and his colleagues performed a yeast two-hybrid screen to identify HSF2-interacting proteins and found that a subunit of condensin enzyme called CAP-G protein binds to HSF2 [63]. HSF2 interacts with the C-terminal domain of CAP-G and binds to the *HSP70* promoter in mitotic cells. They previously found that SUMOylation upregulates the DNA binding activity of HSF2 [64], and the SUMOylation also accelerated HSF2 and CAP-G interaction in mitotic G_2_/M cells. The level of this interaction between SUMOylated HSF2 and CAP-G was higher in G_2_/M cells than G_0_/G_1_ or S cells [63]. These results suggested that HSF2 binds to the *HSP70* promoter in a mitosis-dependent manner and prevents the compaction of this promoter; thus, they hypothesized that HSF2 has an important role in *HSP70* bookmarking.

The bookmarking mechanism was first proposed by Levens’ group [65]. During mitosis, chromatin condenses, transcription is shut off, and most transcription factors are excluded from chromosomes [66,67]. However, several researchers discovered that chromatin is not completely condensed and that chromatin’s structure is disturbed in some genes before Levens’ group’s Nature paper was published [68,69,70]. Levens and his colleagues showed that chromatin conformational distortion of the TATA box region of the *HSP70* promoter occurred specifically during mitosis using the footprinting technique [65], and they showed the same phenomenon on the *c-myc* and beta-globin promoters. To date, bookmarking has been observed in various gene promoters and transcription factors [71,72,73,74]. HSF2 is the first transcription factor to be shown to bind to the gene promoter during mitosis and essential for bookmarking of the *HSP70* gene.

In the bookmarking mechanism on the *HSP70* promoter, interaction between HSF2 and the condensin enzyme CAP-G subunit is required [63]. Sarge and his colleagues discovered that serine–threonine protein phosphatase (PP2A) is recruited to the *HSP70* promoter, and that this recruiting is also necessary for bookmarking [63,75,76]. Condensin activity requires phosphorylation of the CAP-G, CAP-D2, and CAP-H subunits by the mitotic kinase Cdc2–cyclin B complex [77,78]. When condensin interacts with HSF2 via CAP-G, CAP-G is dephosphorylated by PP2A complexed with HSF2. Subsequently, condensin is inactivated and the compaction of DNA on this promoter is prevented [63].

In addition, they discovered that TATA-binding protein (TBP) remains bound to DNA during mitosis and contributes to recruiting PP2A and also that protein regulating cytokinesis 1 (PRC1) also interacts with HSF2 and binds to *HSP70* promoter during mitosis [79,80]. As PRC1 was known to be a CDK substrate that interacts with mitotic spindle and functions in microtubule binding [81,82], they found that HSF2 and PRC1 associate and colocalize during the mitosis phase, whereas PRC1 does not interact with HSF1 [80].

Hayashida reported that HSF2 directly binds to the WD40 repeat protein WDR5, a core component of the Set1 and mixed lineage leukemia (MLL) H3K4 histone methyltransferase complex (Set1/MLL complex), and that HSF2 and major components of the Set1/MLL complex, WDR5, RbBP5, and Ash2L are recruited to the alphaB-crystallin (*CRYAB*) promoter in the same manner as in MEFs [83]. Before this discovery, Vakoc and his colleagues found that MLL1, RbBP5, and Ash2L associate with some gene promoters packaged within condensed mitotic chromosome and thus suggested that the MLL complex also has a mitotic bookmarking function as an additional epigenetic mechanism [84]. The discoveries by Hayashida and Vakoc’s groups may support and confirm the existence of the gene bookmarking function of HSF2.

**Significance in** **Section 3:**

- HSF2 is the first transcription factor shown to have a gene bookmarking function and to bind to the gene promoter during mitosis.
- Chromatin is not completely condensed. The structure is disturbed in some genes.

## 4. Change in HSF1 Expression Level Induces Cell Cycle Arrest

### 4.1. Decreased HSF1 Expression Suppresses Cancer Cell Proliferation

Since a higher expression level of HSF1 in prostate cancer cells and tissue sections from prostate cancer patients was reported, the roles of HSF1 in cancer cells have been examined by many research groups. As already described, overexpression of HSF1 increases the proportion of G_1_ cells, probably by induction of G_1_ arrest, in cervical carcinoma HeLa cells [55]. The effects of reduced HSF1 expression in cancer cells were examined after the report that dominant-negative HSF1 (DN-HSF1) expression inhibits aneuploidy as well as p53 expression in PC-3 p53-null prostate cancer cells [59].

Lindquist and her colleagues showed that cancer cell survival was strongly inhibited by HSF1 knockdown in five human breast cancer BT-20 cells, BT-474 cells, MCF-7 cells, MDA-MB-231 cells, T47D cells, HeLa cells, PC-3 cells, S462 peripheral nerve sheath tumor cells, 90-8 peripheral nerve sheath tumor cells, and 293T in vitro transformed cells, but WI-38 normal fibroblast cells were not affected at all [85]. Muto and his colleagues also reported that HSF1 knockdown prominently reduced the proliferation of human melanoma MeWo cells but not human normal keratinocyte HaCaT cells [86]. These results indicate that reduced HSF1 expression impairs cancer cell proliferation and survival but not normal cell survival and proliferation. Muto and his colleagues also showed that the protein expression of HSP110, HSP90, HSP70, HSP60, and HSP40 was reduced by HSF1 knockdown, but the HSP expression level was not changed in HaCaT cells except HSP90 [86].

A high expression level of various HSPs in human and mouse cancer cells previously reported in the 1980s [87,88,89,90]. The *HSP70* promoter is known to be bookmarked in condensed chromatin during the cell cycle in HeLa cells [63,65], and the HSF–HSP pathway may have more critical roles in cancer cells than in normal cells. We cannot describe here whether both the HSF–HSP pathway and gene bookmarking by HSF are critically important in normal cells; however, recent studies reported that other transcription factors have the gene bookmarking function and that this function has been discovered in normal mammalian cells and in *Caenorhabditis elegans* [91,92,93]. Therefore, the molecular mechanism of the relationship between the HSF–HSP pathway, cell cycle progression, cell canceration, and gene bookmarking is notable and must be further elucidated.

### 4.2. Overexpression of HSF1 Also Causes Suppression of Cancer Cell Proliferation

**Figure 2:**
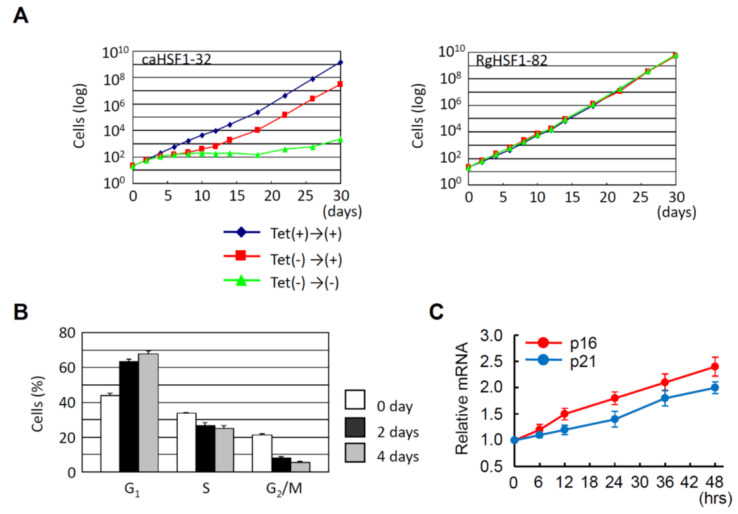
HeLa cell proliferation is inhibited by constitutive active HSF1 (caHSF1). (**A**) caHSF1 expression dramatically inhibits cell proliferation (**left**). In contrast, DNA binding activity lacking mutant HSF1 (RgHSF1) expression does not affect cell proliferation (**right**). (**B**) Change of cell proportion in G_1_, S, and G_2_/M phases by the induction of caHSF1 expression. G_1_ proportion increases and G_2_/M proportion decreases. (**C**) mRNA expression of p16 and p21 increases by the induction of caHSF1 expression.

As already described, overexpression of HSF1 increases the proportion of G_1_ cells in HeLa cells under non-heat-stressed conditions [55]. Hayashida and his colleagues established that the HeLa cells in which constitutive active HSF1 (caHSF1) [94,95] or DNA binding activity-lacking mutant HSF1 (RgHSF1) are inductively expressed using the tet-off system. The caHSF1 lacks a regulatory domain and thus exhibits constitutive transcriptional activity (Figure 1A). RgHSF1 has an amino acid mutation at R71, and this arginine (R) amino acid is replaced by glycine (G). This R71G mutation diminishes DNA binding activity (Figure 1A, 94,95). They discovered that caHSF1-expressing cells proliferate at a very low speed (Figure 2A, left, [96]). In contrast, both RgHSF1-expressing cells and caHSF1 expression-inhibited cells proliferate normally (Figure 2A, right, 96). They examined the cell population and found that G_1_ phase cells were increased 1.5-fold and G_2_/M phase cells decreased by 25% in caHSF1-expressing cells (Figure 2B, 96).

To reveal the mechanism behind this phenomenon, Hayashida and his colleagues examined the expression of p16 and p21 in caHSF1-expressing cells. At 48 h after the start of caHSF1 induction by tetracycline depletion, the expression levels of p16 and p21 were increased 2.3-fold and 1.9-fold, respectively (Figure 2C, 96). It is certain that caHSF1 significantly suppresses cell proliferation though the induction of target genes, but whether p16 and p21 substantially contribute to this phenomenon cannot be identified by only these results. Ilangovan and his colleagues reported that HSP27 binds to p53 and increases p53 transcriptional activity, which increases p21 expression and results in G_2_/M cell cycle arrest [97]. Hayashida and his colleagues previously reported that the expression of most HSPs, including HSP27, increases in the same caHSF1-expressing HeLa cells [95]. One hypothesis is that high expression of HSP27 protein may cause cell cycle arrest and suppress cell proliferation through a similar mechanism. Hayashida’s group showed that caHSF1 expression induces the increased G_1_ cell population (probably through G_1_ cell arrest) [96]. According to these data and previous reports, the effects of HSF1 expression may be more complexed than we imagine. The effects of HSF1 expression have been investigated by many HSF researchers, however, this might be a difficult issue and required to investigated in more detail in HSF research field. 

Finally, it is surprising that both overexpression and knockdown of HSF1 can induce cell cycle arrest and suppress cell proliferation. The molecular mechanisms that cause these contrasting phenomena are notable, and the analysis of these mechanisms may contribute to the discovery of novel functions of HSF1 in cell cycle regulation.

**Significance in** **Section 4:**

- Reduced HSF1 expression (induced by knockdown) inhibits the proliferation of cancer cells but not normal cells.
- Constitutive active HSF1 expression also inhibits the proliferation of cancer cells probably through the induction of G_1_ cell cycle arrest.

## 5. Degradation of HSFs during Cell Cycle

### 5.1. Degradation of Cyclins and CDKs, Subunits of Cyclin-CDK Complex

During the cell cycle, the levels of cyclins and cyclin-dependent kinases (CDKs) are tightly regulated. Several types of cyclin–CDK complexes function during the cell cycle, and the function is indispensable for normal cell cycle progression, appropriate induction of cell cycle arrest, and cell survival. The level of CDKs remains relatively constant, but the level of cyclins oscillates. Cyclins synthesize, bind, and activate CDKs that are then destroyed [98]. Importantly, CDKs cannot be activated without the interaction with cyclins.

As is widely known, proteolytic control in the cell cycle is crucial for cell cycle regulation and is carried out by various cyclin–CDK complexes [99]. In mitosis, the activation of a large ubiquitin-protein ligase, the anaphase-promoting complex (APC, also known as cyclosome and thus frequently described as APC/C) is required for anaphase initiation and exit from mitosis [100]. During anaphase, replicated chromosomes are split and daughter chromatids move to opposite ends of the cell, and the cells proceed to telophase, the last phase. CDC20 and CDH1 (Clb cyclins by dephosphorylating the APC-specificity factor, homolog of CDC20) are activator proteins of APC [100,101] and its cyclin ubiquitination activity.

APC/C is activated not only in anaphase but also other phases. CDC20 activates APC/C in metaphase and CDH1 does so in telophase, and in the G_1_ phase of proliferating cells [102,103,104,105,106,107,108], and during G_0_ in differentiated cells [109]. The APC/C complex targets and degrades proteins related to cell cycle regulation. In the past 10–15 years, HSFs have been found to be degraded by this APC/C complex. Next, we describe the degradation mechanism of HSFs during the cell cycle.

### 5.2. HSFs Are Degraded by APC/C Complex

As described above, there is growing evidence that HSF1 and HSF2 have important roles in cell cycle regulation. Additionally, whether HSFs and cyclins are degraded by the same mechanism or not has been investigated by many researchers studying HSFs. Among them, Lee and his colleagues reported that HSF1 interacts with CDC20 [110]. This interaction inhibits the association between CDC20 and CDC27, which is one of the canonical subunits of the APC/C complex and is important for mitotic exit and transition into G_1_ phase [111,112,113] and suppresses the phosphorylation and ubiquitination activity of the APC/C complex [110]. Subsequently, they showed that HSF1 is localized to the centromere in mitosis and especially to the spindle poles in metaphase, and that phosphorylated HSF1 undergoes ubiquitin degradation during spindle pole localization [114]. Moreover, they discovered that HSF1 degradation only occurs when HSF1 is phosphorylated, and this phosphorylated HSF1 is released from CDC20. CDC20 binds to the APC/C complex again later. HSF1 is phosphorylated at Ser216 by polo-like kinase (Plk1) in early mitosis, and this phosphorylation is stabilized through interaction with CDC20 [114]. Their observation suggested that the phosphorylated HSF1–CDC20 interaction and subsequent HSF1 degradation may be required for mitotic regulation.

HSF2 is known as the gene bookmarking transcription factor [63]. Sistonen and her colleagues discovered that the APC/C complex ubiquitylates and degrades HSF2 through the interaction as HSF2–CDC20 or HSF2–CDH1 as well as HSF1 [115]. The interaction between HSF2 and the APC/C subunit CDC27 or coactivator CDC20 is enhanced by moderate heat stress, and HSF2 degradation is induced during the acute phase of the heat shock response. Finally, HSF2 is removed from the *HSP70* promoter [115].

Sistonen and her colleagues did not refer to HSF2 phosphorylation, and whether HSF2 is phosphorylated during the cell cycle is uncertain. Sarge and his colleagues reported that HSF2 binds to the PR65 subunit of protein phosphatase 2A (PP2A) and activates PP2A [116]. However, they did not discuss whether HSF2 is dephosphorylated. Phosphorylation is very important for the activation of HSF2 (and HSF1); thus, whether the HSF2 phosphorylation state is stable or not during cell cycle progression needs to be identified.

There are not many papers investigating the molecular mechanism of the degradation of HSF1 and HSF2; thus, we need to further this study and accumulate data. However, the findings of Sistonen’s group and Kevin’s group are notable because they indicate that HSF1 and HSF2 are probably degraded with the same mechanism as cyclins.

**Significance in** **Section 5:**

- Both HSF1 and HSF2 are degraded by the APC/C complex with the same mechanism as cyclins.

## 6. HSFs Are Important for Meiosis

Meiosis is an essential sexual reproduction process, and meiotic failure seriously affects the production of offspring. Meiosis produces haploid cells from diploid parental cells and reduces the chromosome number by half. The regulation of the cell cycle is different from mitosis; the S phase is followed by two rounds of cell division [117].

HSFs are involved in meiosis as well as mitosis. At the early spermatocyte stage, autosomal chromosomes undergo complete synapsis, but X and Y chromosomes can only undergo incomplete synapsis because of their different sizes and shapes. At the pachytene spermatocyte stage in prophase I, X and Y chromosomes are segregated into a subnuclear compartment called the XY body (also called the sex body) [118]. The unsynapsed regions of X and Y chromosomes are condensed and repressed, and HSF1 localizes to the XY body and contributes to gene silencing [119]. HSF1 is transiently expressed in meiotic spermatocytes and haploid round spermatids in mouse testes, and HSF1-deficient male mice show increased morphological abnormalities in sperm heads [119].

HSF1 also has important roles in oogenesis. Christians and her colleagues found that metaphase II arrest is normally induced in HSF1-deficient mature oocytes but discovered ultrastructural abnormalities and dysfunctional mitochondria in intraovarian HSF1-deficient oocytes [120]. She previously discovered that the embryos produced by HSF1-deficient female mice initiate early development and do not survive even in the oviduct of wild-type mice in spite of the fact that ovulated eggs of HSF1-deficient mice show proper metaphase II arrest [121]. The cell division of these embryos was blocked mainly at the one-cell stage when they were produced by mating with wild-type or HSF1 heterozygous male mice [121]. These results show that HSF1 has crucial roles in meiosis for both spermatogenesis and oogenesis.

Two groups reported that meiosis is affected by HSF2 deficiency in male mice [122,123]. In HSF2-deficient male mice, Mezger and her colleagues discovered that seminiferous tubules are vacuolated and that these tubules are devoid of all meiotic spermatocytes and post-meiotic haploid spermatids [122]. In addition, they found that dying cells increase in HSF2-deficient testes and that 90% of these dying cells are spermatocytes at meiotic divisions, especially at pachytene meiotic prophase. A similar observation was reported by Mivechi and her colleagues [123]. These findings indicate that spermatogenesis is disrupted by HSF2 deficiency.

As well as HSF1 deficiency, oogenesis was suggested to be affected by HSF2 deficiency. Mezger and her colleagues found that 40% of the eggs from HSF2-deficient female mice are fragmented or devoid of polar bodies [122]. They also observed ovarian defects, including the presence of large hemorrhagic follicles with a trapped oocyte; however, they did not show clear data indicating the meiotic defects in oogenesis. No other groups have reported that HSF2 is involved in oogenesis; thus, whether oogenesis is affected or regulated by HSF2 remains to be elucidated.

**Significance in** **Section 6:**

- HSF1 and HSF2 are involved in meiosis as well as mitosis.
- Spermatogenesis is affected by both HSF1 and HSF2. It is not clear whether oogenesis is also affected by both HSFs.

## 7. Conclusions and Perspectives

In this review, we briefly described the early essential findings in cell cycle studies and the discovery of the heat shock response and essential functions of HSFs and referred to the important discoveries to date. In mammalian cells, four major HSFs (HSF1–HSF4) have been discovered, but human HSF3 is a pseudogene and mouse HSF3 has not been well studied yet [124]. There are no reports referring to the relationship between HSF3 and cell cycle regulation.

Concerning HSF4, Mivechi and her colleagues reported that HSF4b (major isoform of HSF4 [125]) binds to the promoters of HSPs with the Brahma-related gene (Brg1) ATPase subunit of SWI/SNF chromatin remodeling complex, and that the HSF4b-Brg1 complex is formed only during the G_1_ phase [126]. During the G_1_ phase, the chromatin structure is more accessible to transcriptional regulatory proteins; thus, HSF4b may stimulate the expression of HSPs during the cell cycle. However, whether HSF4b has important roles in cell cycle regulation is yet to be elucidated.

The success of molecular cloning of HSF1 and HSF2 gave rise to new experimental techniques and brought about many new discoveries. For example, C-terminal activation domain-lacking mutant HSF1-bearing yeast cells showed to undergo G_2_/M arrest, and overexpression of HSF1 increased the population of G_1_ cells in HeLa under non-heat-stressed conditions [52,65]. The importance of these results should be noted because these phenomena were observed under non-heat stress. After the success of cloning, several experiments were performed under non-heat-stressed conditions (37 degrees when using mammalian cells); thus, we learned additional information about HSF1 and HSF2. In particular, it was discovered that HSF2 is not activated by heat stress meaning that the success of cloning contributed greatly to the progression of HSF2 research.

p53 is widely recognized as a tumor suppressor gene, and its protein product is also a well-known transcription factor regulating the expression of genes involved in cell cycle arrest, apoptosis, and DNA repair [127,128]. In p53-dependent cell cycle arrest, the cyclin-dependent kinase inhibitor p21 (WAF1) is an important mediator [127,129]. Notably, dominant-negative HSF1 (DN-HSF1) increased p21 expression in p53-null PC-3 prostate carcinoma cells [59]. Robson and his colleagues showed that HSF1 interacts with p53 and that this interaction becomes more stable when the cells are treated with the genotoxic compounds actinomycin D, doxorubicin, or etoposide [130]. In addition, heat or doxorubicin stress induced HSF1 translocation to the nucleus together with p53 and TATA-box binding protein (TBP). They also established p53 knockdown cells and HSF1 knockdown cells and examined whether HSF1 is involved in the expression of p53 target genes. They found that expression of the p53 target genes p21, PUMA, gadd45, and PCNA is reduced, and p21 and PUMA expression in particular is dramatically reduced in both knockdown cells [130]. Further, Hayashida and his colleagues also examined whether p21 and p16 genes are regulated by constitutive active HSF1 in HeLa cells and found increased expression of p21 and p16 and dramatic suppression of cell proliferation [96].

These findings strongly indicate that HSF1 can affect p53 functions directly or indirectly. As it is widely known that p53 has an extremely wide range of effects, HSF1 may affect many biological phenomena more than is currently known. HSF1 knockdown, caHSF1 expression, and normal HSF1 overexpression dramatically reduced cell proliferation [55,86,96]. To understand this paradoxical phenomenon and establish a consistent theory of the molecular mechanism, a more detailed analysis of the formation of HSF1-interacting proteins and various HSF1-containing transcriptional complexes will be required. The analysis should also consider their binding to HSF2.

In this review, we also referred to the important roles of WD40 repeat proteins. Many of these proteins have seven WD domains; the most important function of the WD domain is forming protein–protein interaction [131]. CDC20, CDH1, and other CDC proteins are WD40 repeat proteins [132,133,134,135], and these CDC proteins bind to the central regulatory domain of HSF1 [110] and contribute to HSF1 degradation as subunits or core components of the APC/C complex [110,115]. The central domain of HSF2 is not a regulatory function domain [83], but CDC20 and CDH1 bind to this domain of HSF2 and degrade HSF2 [115]. The WD40 repeat protein WDR5 also binds to the HSF2 central domain and contributes to the formation of the HSF2-Set1/MLL histone H3K4 methyltransferase complex [83]. Investigation of the functional complexes formed through WD domains will be valuable and bring about new discoveries in both the cell cycle and HSF research fields. For example, the expression of caHSF1 shows dramatically reduced proliferation, and caHSF1 lacks the central domain to which CDC20 and CDC27 bind. Paradoxically, reduced cell proliferation is also observed in various HSF1 knockdown cancer cells. Investigation of WD domain-mediated complex formation will contribute to determining why these paradoxical phenomena are observed.

We summarized and discussed the cell cycle regulation by HSFs, especially HSF1 and HSF2. For HSF researchers, the most known functions of HSFs are the heat shock response and the maintenance of protein homeostasis (proteostasis [136]); thus, the information on the role of HSF1 and HSF2 during cell cycle and their degradation mechanism is almost similar to that of cyclins during the cell cycle will be very useful. For cell cycle researchers, HSF1 and HSF2 will be unfamiliar terms. Furthermore, although HSF1 is the most well-known HSF, we propose that the biological significance of HSF2 is greater than is currently known and that the importance of HSF2 research will increase.

To date, several groups have shown that HSF1 and HSF2 are involved in both mitosis and meiosis (especially spermatogenesis). These studies might contribute to the development of medicine and therapy. Male infertility plays a role in about half of infertility cases, almost 30% of which are caused by a male factor alone [137]. Konrad and his colleagues analyzed azoospermic patients with defined spermatogenic defects and examined germ cell numbers in the early phase of spermatogenesis with an emphasis on mitosis–meiosis transition [138]. They classify the deficiencies individually by distinguishing between high and low efficiency of spermatogenesis. The patients with maturation arrest in primary spermatocytes show significantly reduced numbers of spermatogonia. In contrast, the patients without histological abnormalities in spermatogenesis can compensate for the number of spermatogonia with a high efficiency of meiotic entry and showed normal numbers of spermatogonia if they did not show maturation arrest [138]. Konrad’s group were the first to suggest that a compensatory meiosis mechanism exists in human spermatogenesis and that whether maturation arrest occurs in primary spermatocytes or not is critical.

There is a hypothesis that other HSFs in addition to HSF1 and HSF2 may have important roles in spermatogenesis and oogenesis. In 2004, Nakahori and his colleagues reported that a novel HSF called HSFY exists on the human Y chromosome as multicopies [139]. The human *HSFY* gene located on Yq has a long open reading frame containing an HSF-type DNA-binding domain and is similar to the *LW-1* gene on the human X chromosome, with 53% homology for the amino acid sequences of their presumed DNA-binding domain (DBD). Unlike HSF1 and HSF2, the 53% homology of DBD is not enough to bind the same DNA promoter sequence; in addition, the DBD homology between HSFY and HSF1 is lower at 31% [139]. The *HSFY* gene generates three different transcripts by alternative splicing, and the corresponding proteins are 401, 203, and 213 amino acids long [140]. Transcript 1 has been detected in testis, brain, pancreas, and sperm tissue but not in other tissues or in Sertoli cells. Transcripts 2 and 3 have been detected only in testes [140]. The DBD exists in only variant 1, which is 401 amino acids long, and no HSFY target genes have been discovered to date. Nakahari’s group found the deletion of *HSFY* from men with azoospermia or oligospermia, and Foresta and his colleagues found the same deletion in infertile men [139,140]. These results suggest that HSFY may have important roles in human spermatogenesis. HSFs, including HSF1, HSF2, and HSFY, are most likely involved in the meiotic phase of spermatogenesis and oogenesis, but further research of these phenomena is still needed.
