# Expression of Concern: Tokunaga et al. Cell Cycle Regulation by Heat Shock Transcription Factors. *Cells* 2022, *11*, 203

**DOI:** 10.3390/cells15131143

**Published:** 2026-06-24

**Authors:** Cells Editorial Office

**Affiliations:** MDPI, Grosspeteranlage 5, 4052 Basel, Switzerland; cells@mdpi.com

With this notice, the Cells Editorial Office alerts the readers to concerns related to this article [1]. Following publication of this review article, concerns were raised regarding the validity of Figure 2C. The authors were contacted and asked to provide clarification. However, as no response was received from the authors, the concerns could not be conclusively resolved.

The Editorial Board assessed the issue and determined that it does not substantially affect the overall findings presented in this publication.

This Expression of Concern is issued to inform readers of the unresolved concerns regarding Figure 2C. The Editorial Board has no further action planned at this time. However, should additional information become available, further action may be taken in accordance with MDPI’s policies if deemed necessary.

## References

[1] Tokunaga Y., Otsuyama K.-I., Hayashida N. (2022). Cell Cycle Regulation by Heat Shock Transcription Factors. Cells.

